# Vasopressin regulates renal calcium excretion in humans

**DOI:** 10.14814/phy2.12562

**Published:** 2015-11-30

**Authors:** Guillaume Hanouna, Jean-Philippe Haymann, Laurent Baud, Emmanuel Letavernier

**Affiliations:** 1 UMR S 1155, Sorbonne Universités, UPMC Univ Paris 06Paris, France; 2 UMR S 1155, INSERMParis, France; 3 Explorations Fonctionnelles Multidisciplinaires, AP-HPParis, France

**Keywords:** Vasopressin, calcium, kidney, SIADH

## Abstract

Antidiuretic hormone or arginine vasopressin (AVP) increases water reabsorption in the collecting ducts of the kidney. Three decades ago, experimental models have shown that AVP may increase calcium reabsorption in rat kidney. The objective of this study was to assess whether AVP modulates renal calcium excretion in humans. We analyzed calcium, potassium, and sodium fractional excretion in eight patients affected by insipidus diabetes (nephrogenic or central) under acute vasopressin receptor agonist action and in 10 patients undergoing oral water load test affected or not by inappropriate antidiuretic hormone secretion (SIADH). Synthetic V2 receptor agonist (dDAVP) reduced significantly calcium fractional excretion from 1.71% to 0.58% (*P *<* *0.05) in patients with central diabetes insipidus. In patients with nephrogenic diabetes insipidus (resistant to AVP), calcium fractional excretion did not change significantly after injection (0.48–0.68%, *P *= NS). In normal subjects undergoing oral water load test, calcium fractional excretion increased significantly from 1.02% to 2.54% (*P *<* *0.05). Patients affected by SIADH had a high calcium fractional excretion at baseline that remained stable during test from 3.30% to 3.33% (*P *= NS), possibly resulting from a reduced calcium absorption in renal proximal tubule. In both groups, there was a significant correlation between urine output and calcium renal excretion. In humans, dDAVP decreases calcium fractional excretion in the short term. Conversely, water intake, which lowers AVP concentration, increases calcium fractional excretion. The correlation between urine output and calcium excretion suggests that AVP-related antidiuresis increases calcium reabsorption in collecting ducts.

## Introduction

Vasopressin or arginine vasopressin (AVP) or antidiuretic hormone (ADH) is secreted by the neurohypophysis in response to increased blood osmolality (Robertson 2006). Arginine vasopressin increases peripheral vascular resistance and exerts antidiuretic effects, promoting water reabsorption in the collecting duct of the kidney. The hormone binds to G-protein-coupled V2 receptors at the basolateral membrane of collecting duct principal cells, activating adenylate cyclase, cAMP formation, and in turn insertion of aquaporin two water channels at the principal cells apical membrane. In addition to its effects on water reabsorption, AVP binding to V2 receptors modulates electrolytes and urea transports. V2 receptor activation increases sodium reabsorption by principal cells and, at least in rodents, by the thick ascending limb of the loop of Henle (Juul et al. 2014).

Humans may be affected by diseases related to inappropriate secretion of AVP (SIADH), leading to impairment of urine dilution by kidney, increased intracellular volume evidenced by low blood osmolality, and moderate increase in extracellular volume (Decaux et al. 2007). An oral water load test results in the repression of AVP synthesis and appropriate urine dilution (increased free water clearance) in healthy subjects but not in patients affected by SIADH. Conversely, some patients are affected by a defect of AVP secretion by neurohypophysis, referred to as central diabetes insipidus, or by kidney insensitivity to the AVP, referred to as nephrogenic diabetes insipidus. In both cases, urine concentration is impaired. The acute infusion of dDAVP results in appropriate urine concentration (negative free water clearance) in patients affected by central diabetes insipidus, but not in patients affected by nephrogenic diabetes insipidus (Morello and Bichet 2001).

Thirty years ago, Bouby et al. investigated the action of AVP on calcium and magnesium excretion in Brattleboro rats, a model of hereditary hypothalamic diabetes insipidus (Bouby et al. 1984). Rats were studied in the absence and after chronic infusion of dDAVP, a nonpressor analog to AVP which binds to V2 receptor. In this model, dDAVP decreased urinary excretion of calcium and exerted a trophic effect on the thick ascending limb of the loop of Henle. To date, whether AVP modulates calcium renal excretion in humans remains unknown.

To investigate whether AVP modulates calcium fractional excretion by kidney in humans, we measured calcium and creatinine concentration in blood and urine before and 3 h after oral water load test in eight patients affected or not by SIADH. The same measures were performed in 10 patients affected by central or nephrogenic diabetes insipidus before and after dDAVP infusion.

## Subjects and Methods

### Patients and study design

All patients were referred to the Physiology Department of Tenon Hospital in Paris between 2009 and 2013 for suspicion of disorder of urine dilution/SIADH or diabetes insipidus.

Patients were included retrospectively in the study if they had either an oral water load test or dDAVP injection and if serum and urine calcium levels had been assessed before and 3 h after water load or dDAVP injection. Calcium and creatinine were measured in blood and urine altogether with sodium and potassium in routine practice since 2009. According to French legislation, no specific written consent was required since no specific intervention has been performed and all data have been recorded retrospectively and anonymized.

Exclusion criteria included kidney transplantation and chronic kidney disease stage IIIb or more according to the CKD-EPI glomerular filtration rate estimation (less than 45 mL/min per 1.73 m²).

Overall, two transplant recipients and one patient affected by advanced CKD have been excluded. At last, 18 patients have been included in the study. Eight of them received dDAVP and 10 have been referred for an oral water load test.

### Methods

Patients suspected to have SIADH did not drink during the 12 h preceding the test and received an oral water load (20 mL/kg) in the Physiology unit. The inability to dilute appropriately urine or SIADH was defined by the persistence of a negative or low (below 1 mL/min) free water clearance after water load test.

Patients with diabetes insipidus were asked not to drink during the 8 h preceding the test and received 4 *μ*g dDAVP intravenously. Central diabetes insipidus was defined by an appropriate urine concentration after dDAVP (i.e., negative free water clearance). Conversely, nephrogenic diabetes insipidus was defined by an inappropriate urine concentration after dDAVP (positive free water clearance).

Urine collection and blood samples were collected in the unit before and every hour (during 3–4 h) after dDAVP injection or water load test. Serum and urine calcium levels were measured before and 3 h after dDAVP injection or water load.

The following parameters have been recorded: diuresis volume and urine flow rate, urine and serum calcium, creatinine, sodium, potassium, and osmolality.

Urinary and plasmatic osmolality were measured with an Advanced Instruments model 3320 osmometer. Sodium, potassium, enzymatic, and Jaffe creatinine were measured with a Konelab 20 Analyzer from Thermo Fisher Scientific. Sodium and potassium were measured with an ABL 815 from Radiometer. Calcium and magnesium were measured with a Perkin Elmer 3300 atomic absorption spectrometer. Plasma levels of AVP were determined in duplicate by radioimmunoassay in patients affected by diabetes insipidus, before the test (Caillens et al. 1982).

Sodium fractional excretion was calculated as following: (urinary sodium × serum creatinine)/(serum sodium × urinary creatinine). Potassium fractional excretion was calculated in the same way. To assess calcium fractional excretion, we considered that ultrafilterable calcium (ionized calcium + calcium in complexes with phosphate, carbonate, lactate, or citrate) was half of the total serum calcium concentration and calculated as following: (urinary calcium × serum creatinine)/(serum calcium × 0.5 ×  urinary creatinine).

Osmolar clearance was calculated on each time period using the following formula: (urinary osmolality × urine flow rate)/plasmatic osmolality.

Free water clearance that represents the volume of pure water subtracted or added to the plasma per unit time was calculated by the formula: urine flow rate − osmolar clearance.

### Statistical analysis

Data are expressed as medians. Unpaired variables were compared by using Mann–Whitney and chi-square tests, whereas paired variables (comparison after/before test) were analyzed by the Friedman test.

The Spearman’s rank correlation test was used for correlations. A *P*-value of less than 0.05 was considered significant. Statistics were performed using Statview® and Graphpad Prism® software programs.

## Results

In all, 18 patients fulfilled inclusion and exclusion criteria. Patient characteristics are summarized in Table1.

**Table 1:** Patient characteristics in the four groups

Patients characteristics	Gender	Age, Years	Weight, kg	Size, m	BMI, kg/m^2^	P Creat μmol/L	CKD-EP mL/min/1.73m^2^	Diagnosis
Group l: Central diabetes insipidus								
Patient 1	M	34	99	1.77	31.6	120	68	Central diabetes insipidus (Histiocytosis)
Patient 2	F	62	82	1.67	29.4	96	55	Partial central diabetes insipidus
Patient 3	M	14	47	1.69	16.5	43	165	Central diabetes insipidus (Pituitary Adenoma)
Patient 4	F	24	66	1.76	21.3	88	79	Central diabetes insipidus
Group 2: Nephrogenic diabetes insipidus								
Patient 5	M	69	72	1.67	25.8	91	72	Nephrogenic diabetes insipidus (Lithium)
Patient 6	M	21	56	1.7	19.4	102	90	Nephrogenic diabetes insipidus
Patient 7	F	34	68	1.67	24.4	113	55	Nephrogenic diabetes insipidus
Patient 8	F	31	57	1.48	26.0	62	116	Nephrogenic diabetes insipidus
Group 3: Normal response to dilution								
Patient 9	M	74	61	1.69	21.4	85	77	Past history of SIADH
Patient 10	M	51	66	1.74	21.8	52	117	Past history of SIADH
Patient 11	F	27	51	1.67	18.3	69	104	Past history of water intoxication
Patient 12	F	37	94	1.67	33.7	56	115	Past history of SIADH
Group4:SIADH								
Patient 13	M	75	89	1.77	28.4	90	72	SIADH
Patient 14	M	67	84	1.67	30.1	83	84	SIADH
Patient 15	F	74	53	1.45	25.2	68	76	SIADH
Patient 16	F	67	72	1.57	29.2	104	48	SIADH (partial response)
Patient 17	M	73	60	1.65	22.0	66	100	SIADH (partial response)
Patient 18	M	81	63	1.59	24.9	84	75	SIADH (partial response)

BMI, body mass index, CKD-EPI, chronic kidney disease – epidemiology collaboration formula; SIADH, syndrome of inappropriate antidiuretic hormone secretion.

### dDAVP injection tests

Patients receiving dDAVP were divided into two groups according to their renal response to dDAVP. Four patients had a negative water clearance after dDAVP injection (median: −0.47 mL/min), and were therefore considered as affected by central diabetes insipidus (Table2). Four patients did not respond to dDAVP, defining a nephrogenic diabetes insipidus (median free water clearance after dDAVP: +0.75 mL/min). Arginine vasopressin dosages have been performed at baseline and were high in patients with nephrogenic diabetes insipidus and low in patients affected by central diabetes insipidus (median: 12.3 and 2.0 pg/mL, respectively, *P *=* *0.03).

**Table 2:** Plasmatic and urinary osmolality and free water clearance before and after dDAVP injection in patients affected by central and nephrogenic diabetes insipidus

	Baseline				Post dDAVP (H3)				Diagnosis
	P Na^+^	P Osm	U Osm	Free H2O Cl. mL/min	P Na^+^	P Osm	U Osm	Free H2O Cl. mL/min	
Group l: Central diabetes insipidus									
Patient 1	145	307	164	0.38	146	307	462	−0.67	Central diabetes insipidus (Histiocytosis)
Patient 2	144	305	323	−0.13	146	306	638	−0.55	Partial central diabetes insipidus
Patient 3	141	291	112	3.32	144	295	369	−0.3	Central diabetes insipidus (Pituitary Adenoma)
Patient 4	146	302	288	0.13	146	299	365	−0.39	Central diabetes insipidus
Median	144.5	303.5	226	0.38	146	302.5	415.5	−0.47	
Group 2: Nephrogenic diabetes insipidus									
Patient 5	145	297	131	1.63	145	292	194	0.61	Nephrogenic diabetes insipidus (Lithium)
Patient 6	141	292	144	0.73	142	297	183	0.9	Nephrogenic diabetes insipidus
Patient 7	146	296	288	2.05	147	307	337	2.46	Nephrogenic diabetes insipidus
Patient 8	143	293	122	0.79	146	309	140	0.57	Nephrogenic diabetes insipidus
Median	144	294.5	137.5	1.21	145.5	302	188.5	0.76	
*P*	1	0.34	0.56	0.34	1	1	0.03	0.03	

P Na^+^, sodium serum level; P Osm, plasmatic osmolality; U Osm, urinary osmolality; free H_2_O Cl, free water clearance.

**Figure 1:**
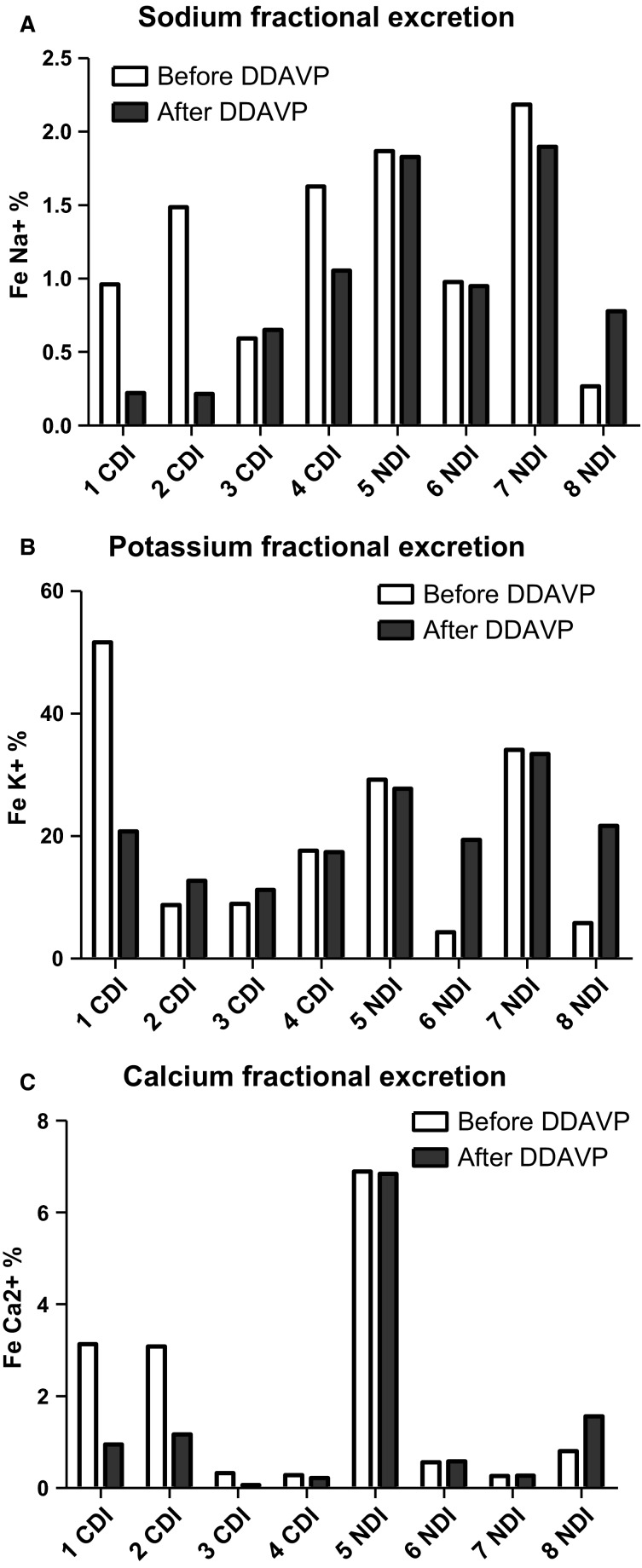
Sodium, potassium, and calcium fractional excretion in patients affected by central diabetes insipidus or nephrogenic diabetes insipidus before (white bars) and 3 h after (dark bars) dDAVP. CDI, central diabetes insipidus; NDI, nephrogenic diabetes insipidus; Fe, fractional excretion.

There was a nonsignificant trend toward a decrease in the median sodium fractional excretion after dDAVP injection in patients affected by central diabetes insipidus, from 1.22% to 0.44% (*P *=* *0.32) and remained stable in patients affected by nephrogenic diabetes insipidus (1.42% to 1.39%, *P *=* *0.32, Fig.1). In parallel, potassium fractional excretion was not significantly modified by dDAVP in both groups (13.3–15.1% and 17.5–24.7%, respectively, *P *=* *0.9, Fig.1). dDAVP reduced significantly calcium fractional excretion from 1.71% to 0.58% (*P *=* *0.046) 3 h after injection in patients with central diabetes insipidus. By contrast, in patients with nephrogenic diabetes insipidus, calcium fractional excretion did not change significantly after injection (0.68–1.07%, *P *=* *0.32, Fig.1).

### Water load test

Patients exposed to oral dilution/water load test were divided into two groups according to their renal response to oral water load. Four “patients” or subjects had a normal response with strong increase in free water clearance (median 4 mL/min), three of them had a past medical history of SIADH attributed retrospectively to drugs or infectious disease, and one had a past history of water intoxication in a context of hypovolemic shock (Table3). Six patients had low free water clearance 3 h after oral load test (median 0.02 mL/min) and were diagnosed SIADH.

**Table 3:** Plasmatic and urinary osmolality and free water clearance before and after dilution/oral water load test in patients affected by SIADH or with a normal response (defined a posteriori)

	Baseline				Post dilution test (H3)				Diagnosis
	P Na^+^	P Osm	U Osm	Free H2O Cl. mL/min	PNa^+^	P Osm	U Osm	Free H2O Cl. mL/min	
Group 3: Normal response to dilution									
Patient 9	137	296	794	−1.07	133	285	201	1.34	Past history of SIADH
Patient 10	139	290	885	−2.77	138	286	132	3.97	Past history of SIADH
Patient 11	140	291	313	−0.15	139	285	104	4.03	Past history of water intoxication
Patient 12	137	286	915	−1.4	135	284	75	6.5	Past history of SIADH
Median	138	290.5	839.5	−1.23	136.5	285	118	4	
Group 4:SIADH									
Patient 13	132	277	460	0.59	127	268	591	0.28	SIADH
Patient 14	130	282	403	−0.76	130	277	613	−0.47	SIADH
Patient 15	132	275	322	−0.5	132	274	267	0.07	SIADH
Patient 16	139	306	580	−0.73	133	290	268	0.14	SIADH (partial response)
Patient 17	135	286	556	−1.75	133	274	308	−0.42	SIADH (partial response)
Patient 18	135	291	369	−0.6	130	277	281	−0.03	SIADH (partial response)
Median	133.5	284	431.5	−0.67	131	275.5	294.5	0.02	
D	0.05	0.33	0.26	0.35	0.02	0.11	0.01	0.01	

P Na^+^, sodium serum level; P Osm, plasmatic osmolality; U Osm, urinary osmolality; Free H_2_O Cl, free water clearance; SIADH, syndrome of inappropriate antidiuretic hormone secretion.

**Figure 2:**
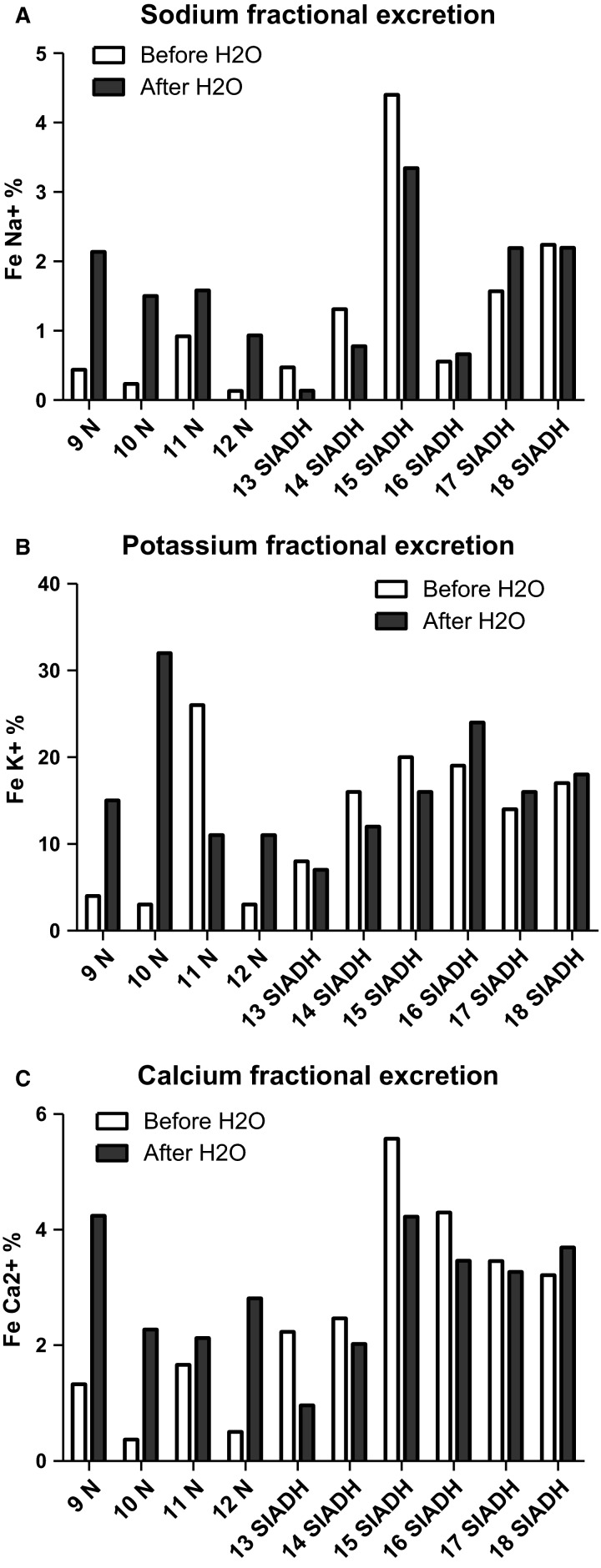
Sodium, potassium, and calcium fractional excretion in patients affected by SIADH or with a normal response before (white bars) and 3 h after (dark bars) dilution test/oral water load. N, normal; SIADH, syndrome of inappropriate antidiuretic hormone secretion.

Sodium fractional excretion increased significantly from a median of 0.34% to 1.54% (*P *=* *0.046) after the oral dilution test in normal subjects and remained stable in patients affected by SIADH (1.44–1.49%, *P *=* *0.9, Fig.2). In parallel, potassium fractional excretion did not increase significantly in normal subjects (11.36–11.48%, *P *=* *0.32), and remained stable in patients affected by SIADH (16.7–15.1%, *P *=* *0.9, Fig.2). Water load increased significantly calcium fractional excretion in normal subjects, from 1.02% to 2.54% (*P *=* *0.046). Calcium fractional excretion was surprisingly high at baseline and remained elevated after water load test in patients affected by SIADH, 3.33% and 3.30%, respectively (*P *=* *0.41, Fig.2).

**Figure 3:**
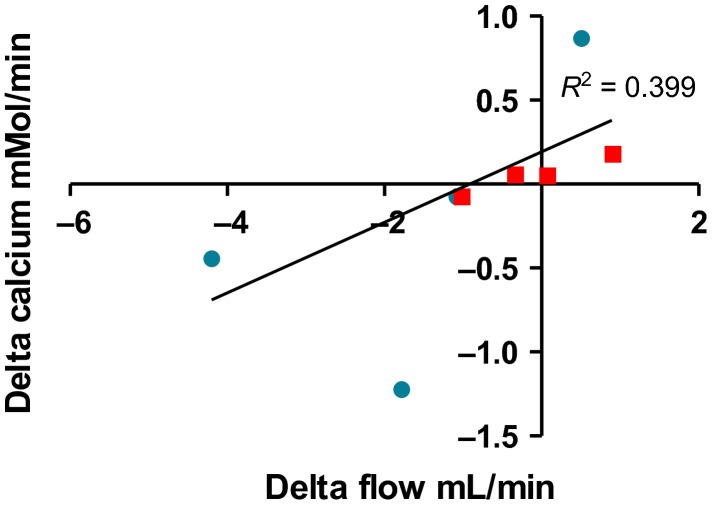
Variation in urine flow rate (*x*-axis, mL/min) and calcium excretion (*y*-axis, mmol/L/min) after dDAVP injection in patients affected by central diabetes insipidus (circles) or by nephrogenic diabetes insipidus (squares).

**Figure 4:**
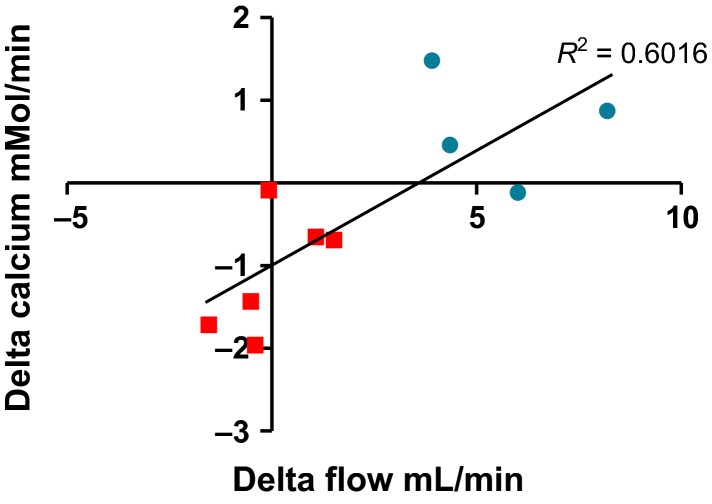
Variation in urine flow rate (*x*-axis, mL/min) and calcium excretion (*y*-axis, mmol/L/min) after dilution/oral water load test in patients affected by SIADH (squares) or with a normal response (circles).

At last, we analyzed whether the variation of urine output (delta after–before test, mL/min) was correlated with the variation of calcium excretion (delta after–before test, mmol/min). After dDAVP injection and dilution test, there was a significant correlation between the variation in urine flow rate and the variation in calcium excretion (*P *=* *0.005 and *P *=* *0.01, respectively, Figs.3 and 4).

## Discussion

Thirty years after the demonstration that AVP (or dDAVP) decreases renal calcium excretion in rodents, these results demonstrate that AVP regulates renal calcium excretion in humans, at least in a short-term manner. Actually, dDAVP decreases renal calcium excretion whereas water load, which decreases acutely plasma osmolality and thereby AVP secretion, increases renal calcium excretion in normal subjects. This regulation is lost in patients whose nephron is unable to respond to AVP (nephrogenic diabetes insipidus) or affected by sustained AVP secretion and water retention (SIADH). These results also confirm to a lesser extent the well-recognized role of AVP in renal sodium reabsorption (Robertson 2006). The seminal experiments performed in rodents highlighted that AVP would increase calcium renal reabsorption through an activation of the V2 receptor in the ascending Henle’s loop (Bouby et al. 1984). In humans, several lines of evidence support the hypothesis that AVP regulates calcium reabsorption in the collecting duct. Almost 30 years ago, Morel et al. demonstrated a response of adenylate cyclase to AVP in the collecting duct from rats, humans, and other mammals. However, unlike rodents, there was no response to AVP in humans Henle’s loop (Morel et al. 1987). Of notice, the presence of V2 receptor in human ascending Henle’s loop remains still debated. Camosino et al. found that V2 mRNA was predominantly expressed in human and mouse collecting duct and failed to find evidence for the expression of V2 mRNA in Henle’s loop thick limb (Carmosino et al. 2007). By contrast, during the same period, Mutig et al. evidenced the presence of V2 mRNA in human Henle’s loop thick limb (Mutig et al. 2007). In addition, micropuncture experiments have shown that AVP stimulates calcium reabsorption in the rabbit cortical collecting system (van Baal et al. 1996). At last, we observed a strong correlation between the delta of urine output occurring after water load test or dDAVP injection and the delta of calcium renal excretion. Although we cannot exclude that vasopressin may play a role in human Henle’s loop, these correlations suggest that kidney modulates calcium reabsorption in collecting duct, the distal part of the tubule responsible for urine concentration. The mechanisms underlying the relationships between urine flow rate in the distal part of the nephron and calcium transport remain elusive. Although the paracellular and transcellular mechanisms responsible for calcium reabsorption in the proximal tubule, in the ascending Henle’s loop and in the distal tubule have been extensively studied, little is known about calcium renal transport in the collecting duct. A channel belonging to the family of the transient receptor potential cation channels, TRPC3, has been recently described in the apical membrane of principal cells in rat (Goel et al. 2006, 2007). AVP induces the membrane trafficking of both TRPC3 and aquaporin two channels through cyclic AMP synthesis in rat collecting duct principal cells, increasing both water and calcium reabsorption (Goel et al. 2007, 2010). TRPC3 is also expressed in human collecting duct, deserving further studies to assess whether AVP regulates calcium reabsorption through TRPC3 in human principal cells or indirectly through urine flow rate. (Letavernier et al. 2012).

The involvement of the collecting duct in calcium homeostasis may be of interest in the field of urolithiasis. Actually, the coupling between water and calcium reabsorption makes sense in a finalist perspective, reducing the risk of crystal and stone formation during antidiuresis. In a similar way, the activation of a calcium-sensing receptor on the apical membranes of collecting duct cells reduces aquaporin-2 expression in animals and probably in humans, limiting water reabsorption and the risk of calcium precipitation in renal tubules (Procino et al. 2012).

Interestingly, patients affected by SIADH have a “paradoxically” high calcium fractional excretion at baseline. Consistent with the former observation, increased daily calcium excretion has also been reported in children and adults affected by nocturnal polyuria and receiving 0.1–0.2 mg/day oral dDAVP, which acts in longer term than intravenous dDAVP, during a mean of 2.13 (range 1–5) days (Chang et al. 2007). SIADH is currently considered as potentially responsible for bone demineralization and osteoporosis and two likely hypotheses have been proposed. First, hyponatremia itself could increase bone resorption and stimulate osteoclast activity (Sejling et al. 2012; Tamma et al. 2013). Second, the moderately increased extracellular volume due to sustained AVP secretion (or potentially dDAVP intake) decreases sodium reabsorption in the nephron proximal tubule, which is coupled with the reabsorption of calcium. The latter hypothesis is supported by the fact that in volume depletion hyponatremia, calcium clearance is normal, whereas calcium fractional excretion is increased in SIADH patients (Decaux et al. 1982). The relatively low calcium serum level at baseline in patients affected by SIADH advocates for a renal calcium leak (median: 2.19 mmol/L). Similarly, patients affected by SIADH have low uric acid serum levels with increased fractional excretion due to low reabsorption in the proximal tubule, a consequence of their relatively high extracellular volume (Decaux and Musch 2008). Taken together, these observations highlight that (dD)AVP decreases calcium fractional excretion in the short term, possibly through a cyclic AMP-dependent pathway in collecting duct cells, whereas sustained elevation of (dD)AVP increases calcium fractional excretion through indirect process. To the best of our knowledge, whether calcium homeostasis is modified in patients affected by central diabetes insipidus remains unknown.

Our results suffer from several limitations. First, the number of patients is relatively small in each group, limiting study power. This problem has been partly solved by using patients as their own controls (comparison after vs. before test), but we may have failed to detect significant decrease in sodium excretion after dDAVP for instance, due to the small size of the groups. Nevertheless, further studies including more patients are required and the analysis of calcium renal excretion in SIADH patients receiving V2 receptor antagonists would also be of interest. In addition, data relative to magnesium excretion have not been analyzed since magnesium dosage has not been performed systematically and we are unable to draw any definitive conclusion on the impact of AVP on magnesium renal excretion.

In conclusion, this study evidences that in the short term, AVP increases renal calcium reabsorption through V2 receptors, probably in the collecting duct, whereas AVP suppression by water intake decreases renal calcium reabsorption. These effects are opposed to those of sustained AVP synthesis or long-term dDAVP intake, which are associated to increased renal calcium excretion through indirect mechanisms involving hyponatremia and/or extracellular volume. These observations deserve potential pathogenic implications in the fields of urolithiasis and bone mineralization and suggest that collecting duct cells could play an unexpected role in calcium homeostasis in humans.

## Conflict of Interest

None declared.
